# Oncogenic MORC2 in cancer development and beyond

**DOI:** 10.1016/j.gendis.2023.05.010

**Published:** 2023-07-03

**Authors:** Shan Zhang, Ayao Guo, Huan Wang, Jia Liu, Chenshuang Dong, Junyi Ren, Guiling Wang

**Affiliations:** a Key Laboratory of Cell Biology, Department of Cell Biology, Ministry of Public Health and Key Laboratory of Medical Cell Biology, Ministry of Education, China Medical University, Shenyang, Liaoning 110122, China; b Department of Breast Surgery, The First Affiliated Hospital of China Medical University, Shenyang, Liaoning 110001, China

**Keywords:** Cancer development, Hallmarks of cancer, MORC2, Therapeutic target, Treatment resistance

## Abstract

Microrchidia CW-type zinc finger 2 (MORC2) is a member of the MORC superfamily of nuclear proteins. Growing evidence has shown that MORC2 not only participates in gene transcription and chromatin remodeling but also plays a key in human disease and tumor development by regulating the expression of downstream oncogenes or tumor suppressors. The present review provides an updated overview of MORC2 in the aspect of cancer hallmark and therapeutic resistance and summarizes its upstream regulators and downstream target genes. This systematic review may provide a favorable theoretical basis for emerging players of MORC2 in tumor development and new insight into the potential clinical application of basic science discoveries in the future.

## Introduction

MORC family CW-type zinc finger 2 (MORC2) is a member of the microrchidia (MORC) superfamily of nuclear proteins with conserved structures, including the GHKL (gyrase, heat shock protein 90 (HSP90), histidine kinase and DNA mismatch repair protein MutL GHKL)-type-ATPase domain, CW-type zinc finger [four cysteines (C) and two tryptophan (W), CW] domain, and Coiled-Coil (CC) domains.^1^, ^2^, ^3^, ^4^ In addition to MORC2, human MORC proteins also include MORC1, MORC3, and MORC4. Previous studies found that MORC1 was specifically expressed in male mouse germ cells and affected male gametogenesis by preventing spermatogenesis during early meiosis.^5^^,^^6^ MORC3, as a human ATPase^7^ and a component of promyelocytic leukemia (PML) nuclear bodies,^8^^,^^9^ is involved in autoimmune disorders.^10^^,^^11^ It was originally considered a potential lymphoma biomarker.^12^ Recent studies found that MORC4, as an oncogene, promotes the growth, migration, and invasion of breast cancer (BC) cells.^13^^,^^14^

It is well established that all four human MORC proteins contain a GHKL-ATPase domain, a CW domain, and several CC domains^1^, ^2^, ^3^, ^4^^,^^7^^,^^15^; however, MORC1 and MORC2 are classified as the I subfamily, while MORC3 and MORC4 belong to the IX subfamily according to the same structural domains they contain.^16^ MORC1 and MORC2 have a CC domain before the CW domain, which is not shown in MORC3 and MORC4.^2^^,^^17^ Meanwhile, MORC2 and MORC3 have a C-terminus CC domain, which is not found in MORC1 and MORC4.^2^ Moreover, MORC2 has a longer GHKL-ATPase domain than other human MORC proteins.^2^ Overall, MORC2 has been shown to contain specific structural characteristics that are associated with its important functions.

It has been shown that MORC2 not only regulates gene transcription^1^^,^^3^^,^^18^^,^^19^ but also promotes chromatin remodeling.^20^, ^21^, ^22^ Besides, growing evidence has shown that MORC2 expression is up-regulated in most cancers^23^ and promotes the growth, metastasis, and metabolic reprogramming of cancer cells.^24^, ^25^, ^26^ Meanwhile, overexpression of MORC2 is also correlated with cancer treatment resistance, including radiotherapy, chemotherapy, and endocrine therapy.^26^, ^27^, ^28^ Moreover, some studies showed that MORC2 performed its function by its upstream regulators and downstream target genes to participate in cancer progression. Therefore, MORC2 has become a new player and promising therapeutic target and is considered a potential diagnostic and therapeutic biomarker of cancer. In the present review, we highlight the role of MORC2 in cancer and provide an outlook on its future in cancer therapy.

## MORC2 is an oncogene

**Figure 1:**
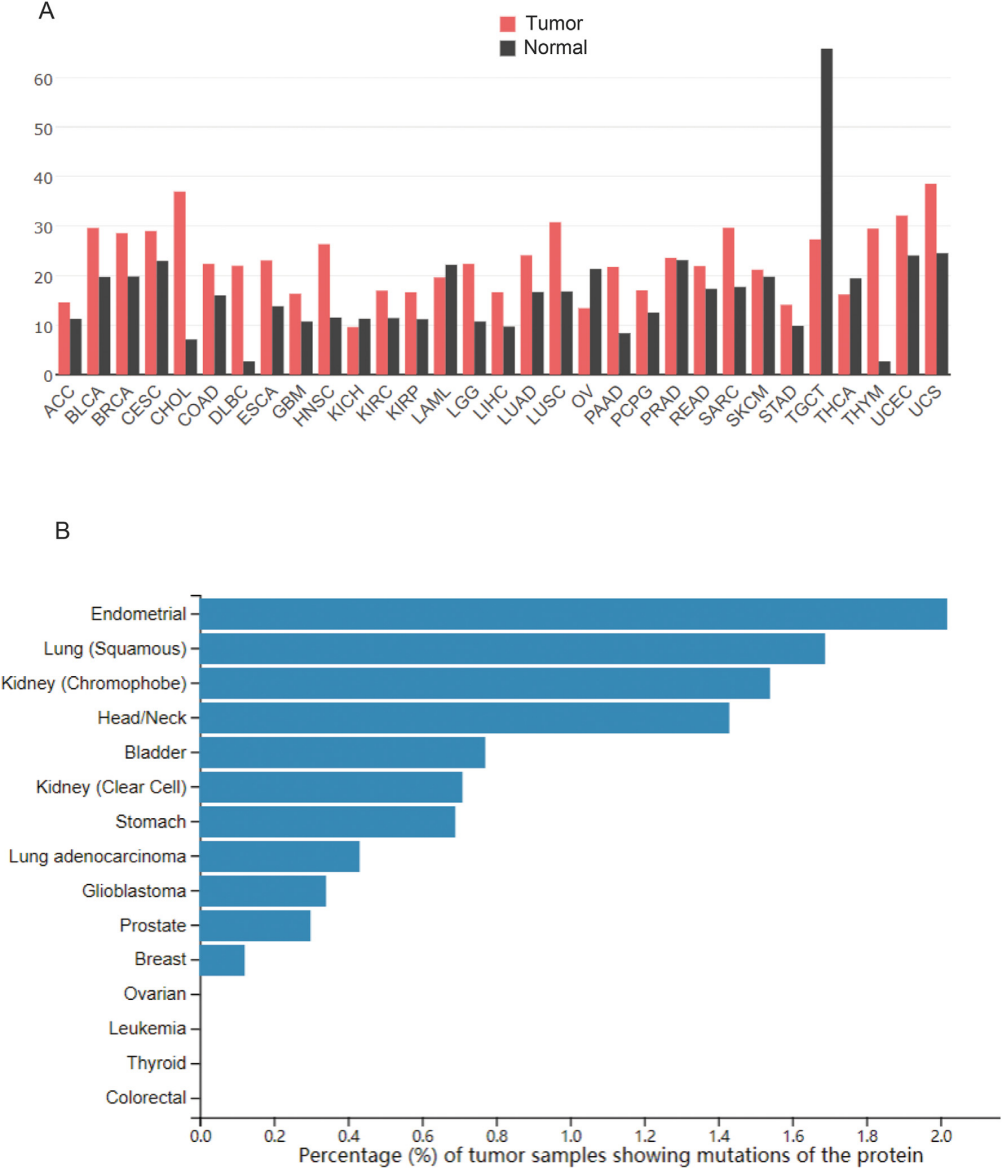
Frequency of MORC2 amplification and mutation in human cancers based on TCGA cancer data using the GEPIA web tool (http://gepia.cancer-pku.cn/). **(A)** The expression profile of MORC2 gene in all tumor samples and paired normal tissues. The height of the bars represents the median expression of a tumor type or normal tissue. **(B)** Frequency of MORC2 protein mutation in cancer. The bar plot above shows the proportion of tumor samples that have any altered mutation in the given protein.

**Figure 2:**
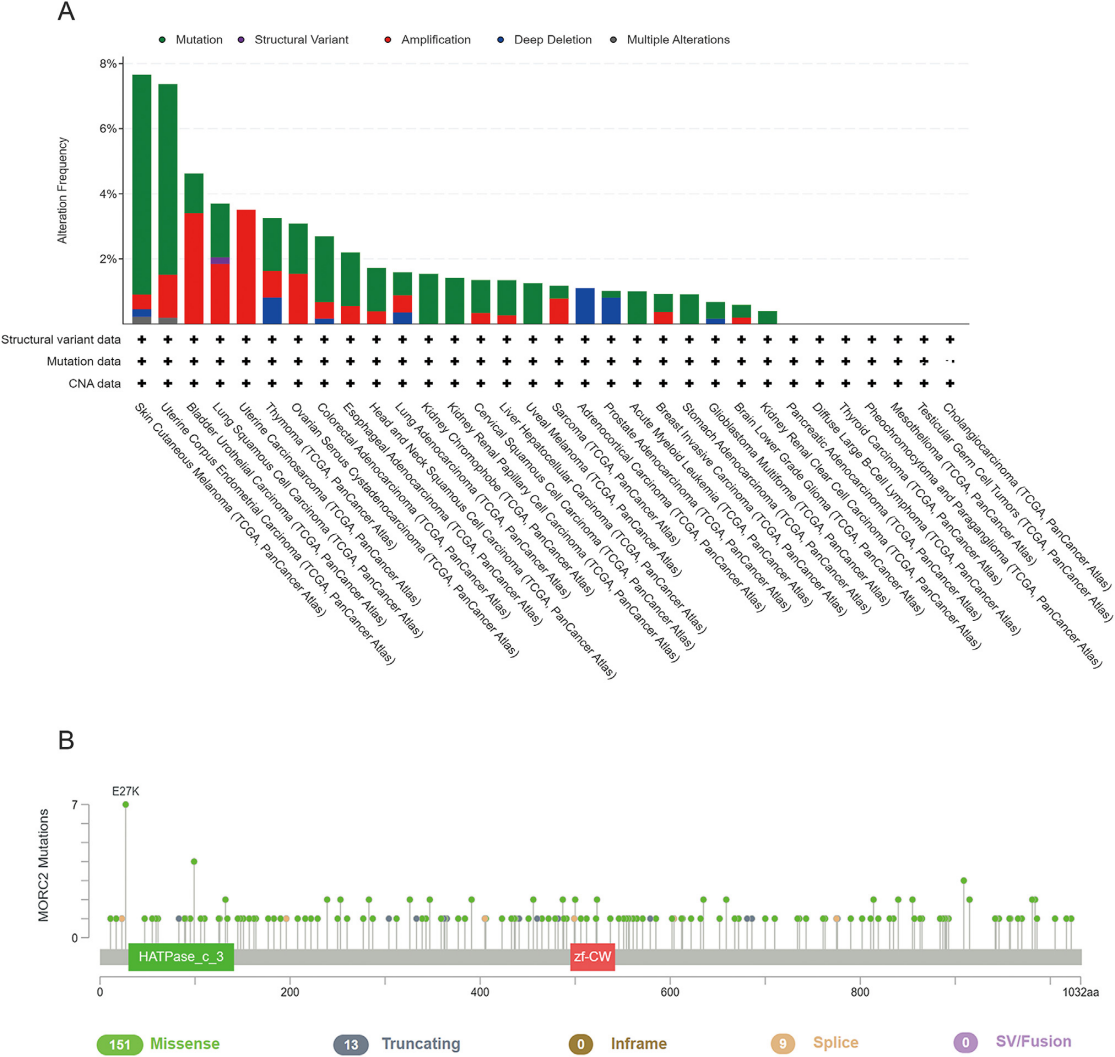
Genetic alteration of MORC2 in human cancer from TCGA cancer data. The data is retrieved using the cBioPortal web tool (http://www.cbioportal.org). **(A)** Genetic alteration of MORC2 in various tumors. **(B)** Frequent MORC2 mutations in human cancers.

Accumulating evidence has shown that aberrations (*e.g.*, amplifications and mutations) in MORC2 are shown in human multiple cancer types (Figure 1, Figure 2), which could be involved in leading to tumor development^23^^,^^26^.

## Amplification

Copy number amplification is a crucial mechanism for oncogene activation and a key step in carcinogenesis.^29^ The cBioportal web tool was used to retrieve The Cancer Genome Atlas (TCGA) cancer data, and it was found that MORC2 was up-regulated in most tumor samples compared with paired normal tissues, exerting its oncogenic activity in various cancers (Fig. 1A). As shown in Figure 2A, MORC2 amplification occurs in most cancers. For example, overexpression of MORC2 promoted the proliferation and invasion of gastric cancer (GC) cells^19^^,^^30^^,^^31^ and hepatocellular carcinoma (HCC) cells.^26^ Similarly, overexpression of MORC2 promoted cholangiocarcinoma cell metastasis through epithelial–mesenchymal transition (EMT) and cell proliferation through serine/threonine kinase (AKT) signaling.^25^ In addition, MORC2 is highly expressed in lung cancer cells and promotes the up-regulation of vascular endothelial growth factor (VEGF) and activation of the Wnt/β-catenin signaling pathway, which triggers the recruitment of tumor-associated macrophages (TAMs) to drive tumor growth.^32^ Furthermore, MORC2 has been shown to interact with histone deacetylase sirtuin 1 (SIRT1) to down-regulate N-myc downstream-regulated gene 1 (NDRG1) as well as promote invasive migration in colorectal cancer (CRC). Moreover, a recent study found that increased expression of MORC2 promotes tumorigenesis by regulating cellular senescence in CRC.^33^

## Mutation

Alteration in the expression levels of associated proteins due to gene mutations can give rise to a significant tumor risk. In the past decade, progress in genome-wide association studies has identified several independent genetic loci of MORC2 for diseases. For instance, MORC2 mutations are associated with some disorders,^34^, ^35^, ^36^ including Charcot-Marie-Tooth (CMT) disease,^37^, ^38^, ^39^, ^40^, ^41^ spinal muscular atrophy,^24^^,^^42^^,^^43^ and neurological disorders,^44^ especially Charcot-Marie-Tooth disease type 2Z (CMT2Z),^38^^,^^45^ thus allowing a better understanding of the genetic architecture of MORC2. Mounting evidence has shown that mutations in MORC2 may be strongly associated with the development of multiple cancers. According to TCGA data, the mutation frequency of MORC2 is relatively high in some cancers (Fig. 1B). MORC2 mutations include missense, truncating, in-frame, splice, and SV/fusion, among which the missense mutation accounts for the largest proportion (87%) (Fig. 2B). M276I of MORC2 increased triple-negative breast cancer (TNBC) invasion and metastasis but did not affect cell proliferation or primary tumor growth. In addition, the M276I mutation enhances hnRNPM-mediated splicing of CD44 from the exon-containing CD44 variant isoform (CD44v) to the exon-absent CD44 standard isoform (CD44s), resulting in EMT and TNBC progression.^24^

## Upstream regulators of MORC2

Increasing evidence confirms that some regulators of biosynthesis (*e.g.*, protein kinase and non-coding RNA/ncRNA) play an important role in cancer progression by regulating MORC2 (Table 1).

**Table 1:** Upstream regulators of MORC2.

Upstream Effectors	Mechanism	Function	Type of Cancer	References
*Proteins*				
PAK1	PAK1 phosphorylates MORC2 to increase ATPase activity and promote cancer cell cycle transition	DNA repair and cell proliferation	breast cancer, gastric cancer	^20^,^31^
PRKACA HSPA8 LAMP2A	GPER1 activates PRKACA to phosphorylate MORC2 and then blocks its interaction with HSPA8 and LAMP2A to stabilize MORC2	Cell proliferation and endocrine resistance	Breast cancer	^28^
c-Myc	c-Myc and MORC2 bind to regulate the transcription of LDHA	Cell glucose metabolism	Breast cancer	^46^
*Non-coding RNAs*				
miR-186-5p	MiR-186-5p activates the Akt signaling pathway and EMT by regulating MORC2 expression	Cell growth and metastasis	Cholangiocarcinoma	^25^
circDNM3OS	CircDNM3OS sponged miR-145-5p to elevate MORC2 expression	Cell growth and glutamine metabolism	Cholangiocarcinoma	^47^

## Protein-mediated MORC2 regulation

It is well known that molecular structures account for functions. Thus, different structural domains of MORC2 interact with different regulatory factors through their signals to regulate MORC2 function.

MORC2 was first reported as a substrate of p21-activated kinase 1 (PAK1) of AKTs.^48^ Following DNA damage, PAK1 is activated to phosphorylate MORC2 at the serine 739 site and subsequently recruited to chromatin, which induces phosphorylation of H2AX (γH2AX) and chromatin remodeling, maintaining genomic integrity, which can lead to efficient post-damage repair.^20^ In addition, PAK1 was overexpressed in GC.^49^, ^50^, ^51^ In the presence of the small GTPase protein Cdc42, PAK1 activation promotes MORC2 phosphorylation at the serine 677 site, which promotes cell cycle transition from the G1 to the S phase, thus inducing the growth and proliferation of GC cells.^31^

In addition, MORC2 was phosphorylated at T582 by protein kinase cAMP-activated catalytic subunit alpha (PRKACA), which is activated by G protein-coupled estrogen receptor 1 (GPER1), enhancing its stability by reducing interaction with heat shock protein family A (Hsp70) member 8 (HSPA8) and lysosome-associated membrane protein 2 (LAMP2A), suggesting that phosphorylation modification of MORC2 plays a role in preventing its degradation by chaperone-mediated autophagy (CMA) lysosomes, thereby resulting in E2-mediated BC cell proliferation via the GPER1-PRKACA-MORC2 pathway.^28^

Reliable evidence has shown that c-Myc plays a vital role in the glucose metabolism of cancer cells, which can promote tumor growth and metastasis.^52^, ^53^, ^54^ Guddeti et al demonstrated that MORC2 is a glucose-inducible gene and serves as a target gene of c-Myc to promote lactate dehydrogenase A (LDHA) transcription and BC cell migration at high glucose concentrations.^46^

## Non-coding RNA-mediated MORC2 regulation

Non-coding RNA is also considered the “dark matter in life” and is involved in regulating various biological functions at the RNA level through diverse mechanisms.^55^ Although studies on different types of ncRNAs, including microRNA (miRNA), long non-coding RNA (lncRNA), circular RNA (circRNA), *etc.*, have flourished in the past two decades,^56^ the regulatory effect of ncRNA on MORC2 regulation has been scarcely reported. Recent studies found that miRNA and circRNA participated in the regulation of MORC2 expression.^25^^,^^47^ However, the role of lncRNA in MORC2 regulation has received less attention.

Numerous studies have shown that miRNAs play a vital role in cell proliferation and death,^57^ as well as a variety of cellular activities.^58^, ^59^, ^60^ Additionally, miRNAs are aberrantly expressed in various types of cancers and can be manipulated to control the growth of cancer cells.^61^, ^62^, ^63^ miRNA has been shown to regulate MORC2 in cancer. For example, miR-186 is a specific cancer-type miRNA. miR-186-5p expression was down-regulated in CRC, and low expression of miR-186-5p leads to the overexpression of MORC2, which promotes the growth and metastasis of cholangiocarcinoma (CCA) cells by regulating Akt signaling and EMT.^25^

circRNAs are a group of endogenous biomolecules with a closed-loop structure that play key biological roles in various diseases, including cancer.^64^^,^^65^ The distinctive structure of circRNAs makes them more resistant to ribonucleic acid exonuclease R (RNase R), making them a hot spot for research on diagnostic biomarkers and therapeutic targets.^66^ MORC2 is a target gene of miR-145-5p, and high expression of circDNM3OS increased MORC2 expression by sponging miR-145-5p in CRC, which in turn promoted the progression of CRC.^47^

## The downstream target genes of MORC2

Similarly, different structural domains of MORC2 exert their function by regulating different downstream target genes (Table 2). Carbonic anhydrase IX (CAIX) — a transmembrane isoenzyme — is the first downstream target gene of MORC2 to be identified. MORC2 binds to protected region 4 (PR4) in the CAIX promoter by recruiting histone deacetylase 4 (HDAC4) to reduce the histone H3 acetylation level, thus resulting in the closure of the chromatin structure and suppressing CAIX transcription to promote GC cell proliferation.^18^^,^^67^^,^^68^ ATP citrate lyase (ACLY) is a major factor in the conversion of glucose from mitochondria to fatty acids in the form of citrate export.^69^ In addition, ACLY expression is up-regulated in different cancer types.^70^, ^71^, ^72^ ACLY-dependent acetyl coenzyme A production for lipogenesis is important for the proliferation of tumor cells.^73^^,^^74^ MORC2 can specifically interact with ACLY in the cytoplasm of BC cells, inducing the activation of ACLY phosphorylation, acetyl-CoA carboxylase (ACC), and fatty acid synthase (FAS), ultimately leading to elevated fatty acid levels and promoting adipogenesis and adipocyte differentiation.^75^

**Table 2:** Downstream target genes of MORC2.

Downstream Effectors	Mechanism	Function	Type of Cancer	Reference
CA Ⅸ	MORC2 binds to CA Ⅸ and represses its transcription by recruiting HDAC4	Cell growth and survival	Gastric cancer	^18^
ACLY	MORC2 interacts with ACLY in the cytoplasm and induces ACLY activation	Adipogenesis and adipocyte differentiation	Breast cancer	^75^
p21	MORC2 binds to p21 and represses its transcription by recruiting HDAC1	Cell proliferation and cell cycle	Gastric cancer	^19^
ArgBP2	MORC2 forms a complex with EZH2 and binds to ArgBP2 to suppress its transcription	Cell proliferation, invasion, and migration	Gastric cancer	^76^,^77^
	MORC2 promotes the binding of PRC2 and EZH2 with HSF1 to ArgBP2 and thus restricts the transcription of ArgBP2			
NF2 KIBRA	MORC2 interacts with DNMT3A and represses the transcription of Hippo signaling regulators NF2 and KIBRA	Cancer stem cell and proliferation	Liver cancer	^78^
CD44	MORC2 M276I enhances MORC2 incorporation with hnRNPM and facilitates the shift of CD44v to CD44s	Cell invasion, migration, and metastasis	Breast cancer	^24^
PARP1	MORC2 activates chromatin remodeling activity upon DNA damage by PARylation and stabilizes PARP1 by acetylation	DNA repair and cell survival	Breast cancer	^79^
NDRG1	MORC2 interacts with SIRT1 and restrains the transcription of NDRG1 gene	Cell invasion, migration, and metastasis	Colorectal cancer	^80^,^81^
	MORC2 binds to NDRG1 to inactivate the promoter and inhibit PTEN/PI3K/AKT signaling	Cell invasion, migration, and EMT	Glioma	
C/EBPα	MORC2 regulates C/EBPα expression by SUMOylation and ubiquitination	Cell differentiation and proliferation	Gastric cancer	^30^
P53	MORC2 inhibition leads to decreased HDAC4 and increased p53 and p21 levels	Cell proliferation and cellular senescence	Colorectal cancer	^33^

p21, an important cell cycle inhibitor, is deemed to be a key regulator of cell proliferation and survival.^82^^,^^83^ Our previous studies showed that MORC2 inhibited the expression of p21 in GC cells by recruiting histone deacetylase 1 (HDAC1) to bind to the p21 promoter. Therefore, MORC2-mediated p21 inhibition promotes the cell cycle progression of GC.^19^ Arg-binding protein 2 (ArgBP2), an adaptor protein, performs an essential function in cell adhesion and migration of actin-dependent processes.^84^ It was shown that MORC2 formed a complex with an enhancer of zeste homolog 2 (EZH2) and bound to the ArgBP2 promoter.^77^ MORC2 also promotes the binding of polycomb repressive complex 2 (PRC2) and EZH2 with heat shock transcription factor 1 (HSF1) to the ArgBP2 enhancer and promoter, which enhances the trimethylation of histone H3 on lysine 27 (H3K27),^76^ and thus the complexes cause ArgBP2 transcription repression and the invasive migration of GC cells.

The Hippo signaling pathway plays a vital role in the control of stem cell maintenance and tumorigenesis in the liver.^85^^,^^86^ Wang et al demonstrated that MORC2 interacted with DNA methyltransferase 3A (DNMT3A) to form a complex upstream of the Hippo regulator neurofibromatosis 2 (NF2) and kidney and brain protein (KIBRA) promoters, leading to their DNA hypermethylation and transcriptional repression, Hippo signaling pathway activation, and cell growth inhibition, with implications for cancer cell stemness and tumorigenesis.^78^

CD44 — a cell surface adhesion molecule — is overexpressed in cancer stem cells (CSC) and used as a CSC marker of BC and other cancer types.^87^ CD44 generates two variable isoforms by undergoing extensive alternative splicing, including CD44v and CD44s.^88^ Isoform switching from CD44v to CD44s is functionally essential for cells to undergo EMT, suggesting that shifting splicing regulates cellular phenotypes between epithelial and mesenchymal states.^89^^,^^90^ In TNBC, the M276I mutation in MORC2 enhances the binding of MORC2 to heterogeneous nuclear ribonucleoprotein M (hnRNPM) and promotes the shifting of CD44 isoforms from CD44v to CD44s to induce the EMT of TNBC, which in turn drives the progression of TNBC metastasis.^24^

Poly(ADP-ribose) polymerase 1 (PARP1), a pivotal chromatin-associated enzyme, enhances chromatin remodeling activity^91^^,^^92^ and can interact with MORC2 to catalyze PARylation of MORC2 after DNA damage, thereby promoting DNA repair.^79^ Contrastingly, MORC2 stabilized PARP1 by strengthening the acetylation of PARP1 at K949, which was mediated by the acetyltransferase N-acetyltransferase 10 (NAT10), thereby preventing its ubiquitination at the same residue and following degradation by the checkpoint with forkhead and ring finger domains (CHFR) of E3 ubiquitin ligase, facilitating DNA repair after damage.

NDRG1 — a well-featured metastasis suppressor — is down-regulated in CRC tissues.^93^ SIRT1, a histone deacetylase, can regulate NDRG1 transcription. Meanwhile, MORC2 can interact with SIRT1 and represses the NDRG1 promoter activity, resulting in reduced acetylation levels of histones H3 and H4 in the NDRG1 promoter, thereby repressing NDRG1 gene transcription and further promoting CRC cell migration and pulmonary metastases.^80^ Besides, MORC2 is up-regulated in glioma cells and binds to the NDRG1 promoter to promote glioma cell growth and metastasis by regulating PTEN/PI3K/AKT signaling.^81^

CCAAT/enhancer-binding proteins (C/EBPs) are a group of transcription factors that can regulate cell proliferation and differentiation in various tissues, among which ubiquitination modification of a family member C/EBPα reduces tumor suppressor activity in various human cancers.^94^^,^^95^ MORC2 has been shown to regulate C/EBPα expression through SUMOylation, achieving a shift in cell differentiation toward a cell proliferation program, which induces tumorigenesis.^30^

p53 and p21 are commonly considered signatures of aging.^96^ It was recently reported that MORC2 was highly expressed and inhibited the p53 activity in CRC cells. Inhibition of MORC2 expression resulted in decreased HDAC4 and increased p21 and p53 levels. Thus, the MORC2-HDAC4 signaling pathway promotes senescence and inhibits CRC proliferation in part by increasing the expression of p21 and p53.^33^

## Hallmarks of MORC2

**Figure 3:**
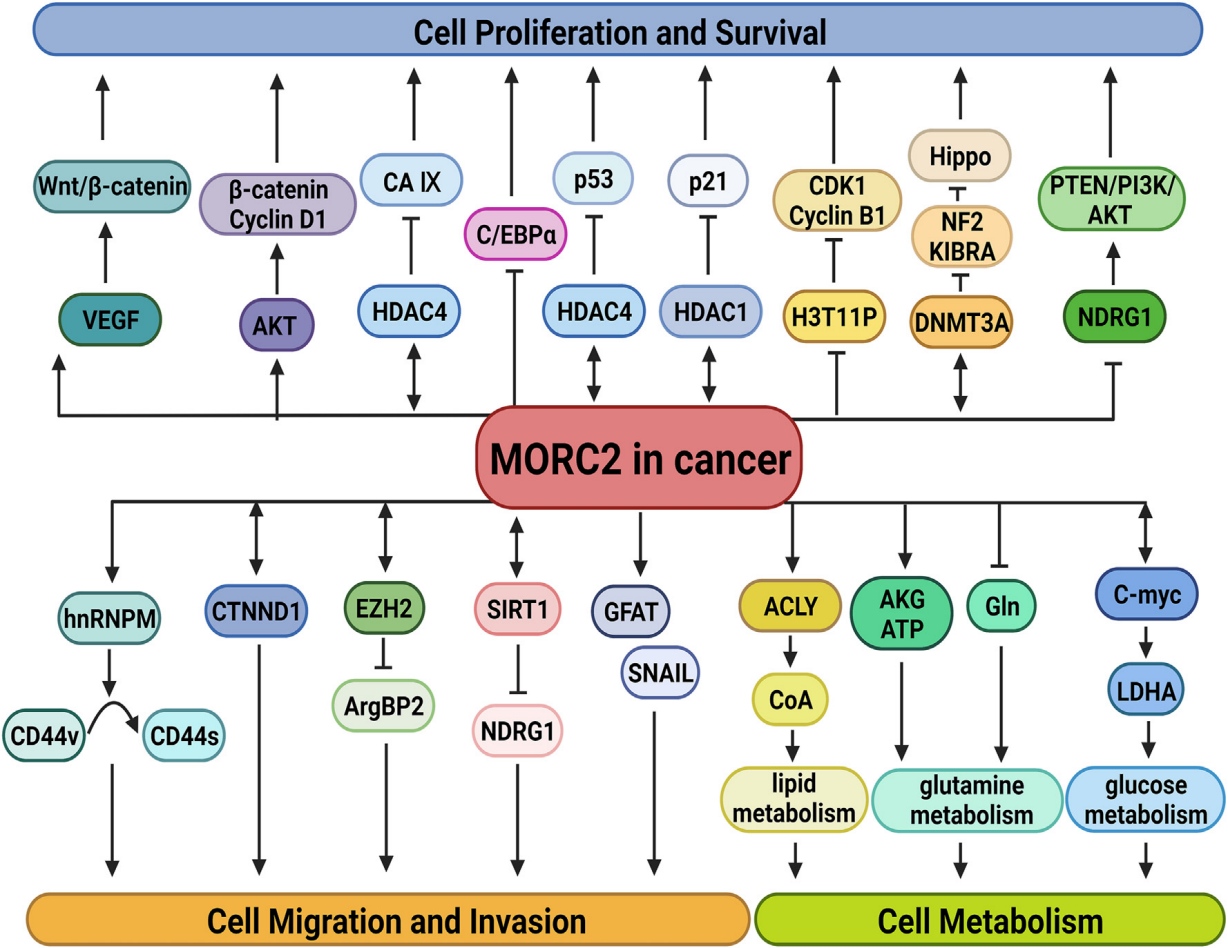
MORC2 is involved in multiple processes of cancer occurrence and development, including cell proliferation and survival, migration and invasion, and cell metabolism.

MORC2 facilitates cancer progression by regulating some aspects of cancer hallmarks demonstrated in cell proliferation, invasion, and migration, as well as cell metabolism. Some of the significant examples are discussed below. The functions of MORC2 in cancer are shown in Figure 3.

## MORC2 promotes tumor cell proliferation and survival

Proper regulations of cell proliferation and survival are required for normal development; however, abnormal cell proliferation and survival may cause tumorigenesis. MORC2, an up-regulated protein, plays a crucial role in promoting tumor proliferation and survival by modulating essential signal pathways, thereby contributing to malignant transformation. It functions as a co-regulator, exerting its effects by either suppressing tumor suppressor genes or promoting oncogenes. Consequently, this dysregulation leads to the aberrant proliferation and survival of cancer cells. Our previous studies showed that MORC2 promoted GC cell proliferation by inhibiting p21 expression. Besides, PAK1 activation induced MORC2 phosphorylation and promoted cell cycle transition from the G1 to S phase of GC, which led to the growth and proliferation of GC cells.^31^ It was reported that MORC2 and HDAC4 complex repressed CAIX transcription and promoted GC cell proliferation and survival.^18^ MORC2 also facilitates cellular differentiation towards a cell proliferation program that induces tumorigenesis by regulating C/EBPα expression. In addition, MORC2 drove tumor growth by activating Wnt/β-catenin signaling.^32^ Moreover, MORC2 acetylation at K767, which is regulated by NAT10, promotes the transcription of cyclin-dependent kinase 1 and cyclin B1 to trigger the activation of the G2 checkpoint, which drives cell survival and progression of BC.^27^

Up-regulated MORC2 significantly enhances the proliferation of HCC cells by disrupting p53 and Hippo signal pathways.^26^ Overexpression of MORC2 promotes CCA cell proliferation by activating the AKT signaling pathway.^25^ Similarly, overexpression of MORC2 has been reported to promote BC cell proliferation by up-regulating the expression of β-catenin and AKT signaling.^97^ In addition, up-regulated MORC2 promotes glioma cell growth by suppressing the NDRG1 promoter activity and regulating PTEN/PI3K/AKT signaling.^81^

## MORC2 enhances tumor cell migration and invasion

Migration and invasion are key events in tumor progression, which contribute to a higher mortality rate and a poor prognosis. In addition to cell proliferation and survival, MORC2 also plays an important role in tumor migration and invasion.

MORC2 can promote the proliferation and invasion of GC cells by forming a complex with EZH2 or HSF1 to inhibit ArgBP2 expression.^76^^,^^77^ Moreover, MORC2 enhances CRC cell metastasis by interacting with SIRT1 to down-regulate NDRG1 expression.^80^ Catenin delta 1 (CTNND1) is an intracellular signaling protein that promotes E-calmodulin-mediated inhibition of tumor invasion and metastasis.^98^, ^99^, ^100^ Increased cytoplasmic localization of CTNND1 was found to be highly correlated with an enhanced invasive phenotype of E-calmodulin-deficient BC. MORC2 can interact with CTNND1 and increase the cytoplasmic localization of CTNND1 to promote BC cell invasion and metastasis.^21^ Likewise, mutant MORC2 M276I can interact with hnTNPM to stimulate the shift of CD44 from epithelial to mesenchymal subtypes, leading to EMT and promoting invasive metastasis of BC cells.^24^ SNAIL is a major regulator of EMT and a robust inducer of BC invasion and metastasis. The connective tissue growth factor (CTGF) also plays a crucial role in the migration, invasion, metastasis, and angiogenesis of human BC cells.^101^, ^102^, ^103^ Recent studies have shown that MORC2 at T556 can be O-GlcNAcylated by O-GlcNAc transferase (OGT). Meanwhile, O-GlcNAcylation of MORC2, which was enhanced by transforming growth factor-β1 (TGF-β1), promoted the metastatic and invasive ability of BC by activating the CTGF and SNAIL transcription of TGF-β1 target genes.^104^

## MORC2 reprograms tumor cell metabolism

To meet the ongoing energy needs of tumors during development, tumor cells have to undergo metabolic reprogramming, which is recognized as an emerging hallmark of cancer.^105^^,^^106^ Some pieces of evidence demonstrated that MORC2 participated in metabolic alterations observed in lipid, glucose, and glutamine metabolism, which support the uncontrolled growth and malignant metastasis of cancer cells.^46^^,^^47^^,^^75^ However, the underlying regulatory mechanisms are still poorly understood.

## MORC2 in lipid metabolism

Lipid metabolism reprogramming is a novel confirmed hallmark of cancer. Tumor cells utilize lipid metabolism to obtain energy and enhance lipogenesis, maintain the structure of biological membranes, and serve as signaling molecules, which are needed for rapid tumor growth and response to cancer therapy.^107^ A previous study found that MORC2 promotes lipogenesis and adipocyte differentiation by interacting with ACLY in the cytoplasm, to induce ACLY activation and increased production of Coenzyme A (CoA),^75^ suggesting that MORC2 is involved in the reprogramming of lipid metabolism.

## MORC2 in glucose metabolism

According to the Warburg effect, cancer cells can depend on glycolysis rather than mitochondrial oxidative phosphorylation to meet their energy demand even in the near absence of oxygen.^108^ Recently, it was found that MORC2 is a glucose-inducible gene and a target gene of c-Myc. Conversely, c-Myc can bind to the MORC2 promoter to regulate its expression in cancer cells at high glucose concentrations. Moreover, MORC2 expression is positively associated with the production of various glycolytic enzymes in BC patients. Furthermore, c-Myc can facilitate the MORC2 level and cooperate with MORC2 to regulate the LDHA expression and LDH enzyme activity via a feed-forward loop mechanism.^46^ Thus, MORC2 has a crucial effect on the glucose metabolic reprogramming of BC cells.

## MORC2 in glutamine metabolism

Glutamine metabolism is a cancer hallmark serving as the main energy support to promote cell proliferation and survival in cancer cells.^105^^,^^109^ In particular, most malignant tumor cells always exhibit glutamine addiction.^110^ Mounting studies showed that ncRNAs were involved in metabolic reprogramming^111^^,^^112^; however, the role of ncRNAs in MORC2 regulation in cell metabolism is yet to be deciphered.

Interestingly, recent studies found that circDNM3OS up-regulates MORC2 expression and increases glutamine consumption by sponging miR-145-5p to regulate glutamine metabolism in CCA cells.^47^ With the in-depth study of MORC2, many ncRNAs regulating MORC2 function, including cell metabolism, will be discovered.

## MORC2 drives cancer therapeutic resistance

**Figure 4:**
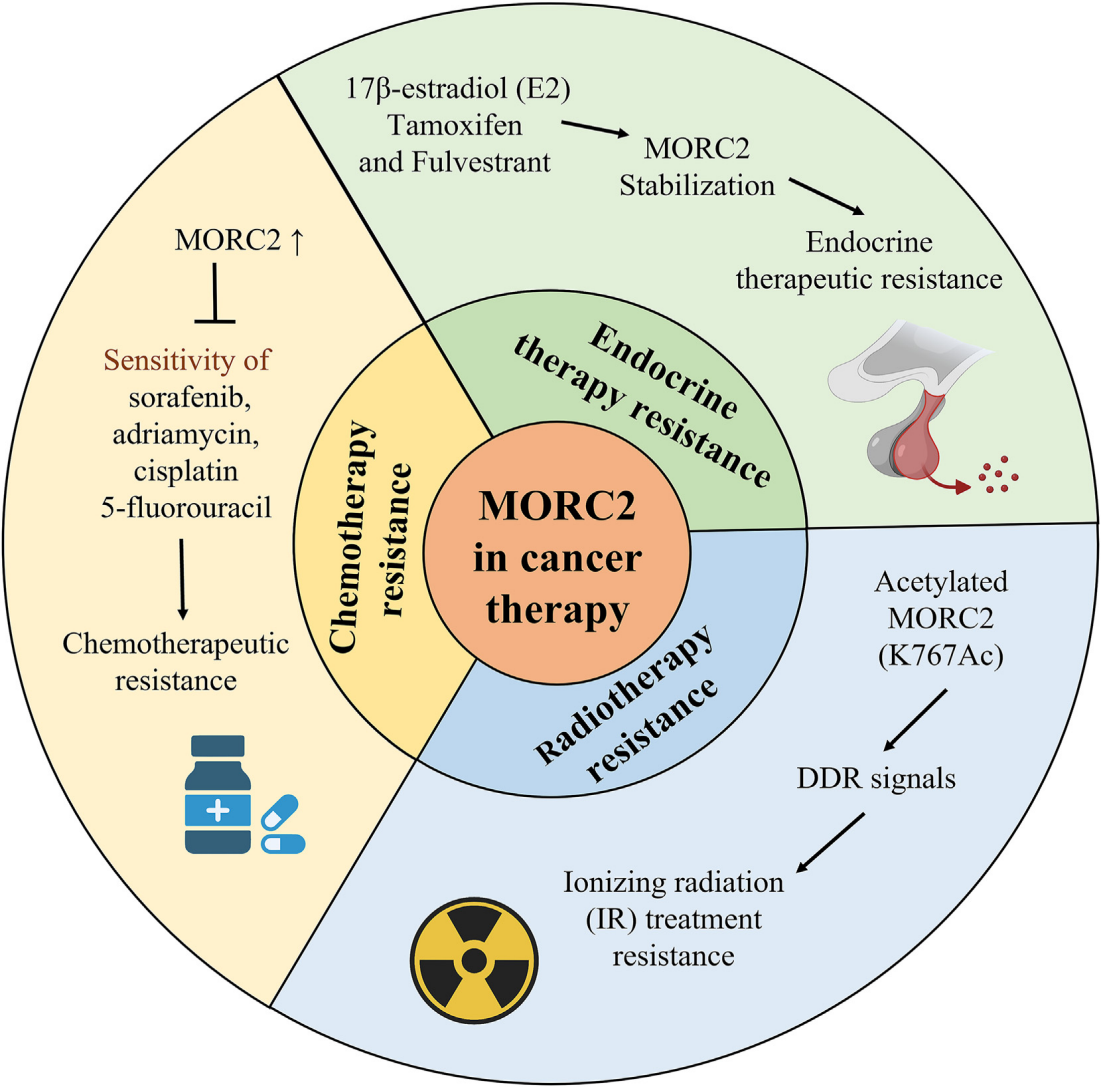
MORC2 is associated with cancer treatment resistance including radiotherapy, chemotherapy, and endocrine therapy.

Several studies found that overexpression of MORC2 in cancer enhances therapeutic resistance, including radiotherapy, chemotherapy, and endocrine therapy^26^, ^27^, ^28^ (Fig. 4).

## MORC2 in radiotherapy

Recent studies have found that the acetylation level at the K767 site (K767Ac) of MORC2 was up-regulated after ionizing radiation (IR) treatment, suggesting that MORC2-K767Ac, as a mediator of DNA damage and repair signals, activates the G2 checkpoint, maintains genomic integrity, and promotes cell survival after exposure to IR.^27^

## MORC2 in chemotherapy

It has been previously found that decreased MORC2 expression increased the sensitivity of HCC cells to the three most common chemotherapeutic agents, including doxorubicin, cisplatin, and 5-fluorouracil.^26^ Conversely, the up-regulation of MORC2 expression led to the resistance of HCC cells to chemotherapy drugs. Meanwhile, the inhibition of the GHKL-ATPase activity of MORC2 enhanced the sensitivity of HCC cells to sorafenib by regulating the Hippo signaling pathway,^78^ suggesting that inhibition of targeted GHKL-ATPase activity of MORC2 is a promising strategy to prevent chemotherapy resistance.

Subsequently, 17-allylamino-17-demethoxy-geldanamycin (17-AAG), an N-terminal inhibitor of HSP90, by selecting many drugs for treating BC was found to effectively inhibit the growth and metastasis of MORC2-overexpressing BC. MORC2 has a GHKL-ATPase domain, which contains the HSP90 protein. Remarkably, 17-AAG can promote MORC2 autophagic degradation by breaking its N-terminal homodimer formation but not affecting its HSP90 expression and ATPase activities.^113^ Therefore, 17-AAG as an HSP90 inhibitor is independent of HSP90 expression in cells and MORC2 ATPase activity.

In addition, a recent study reported that SUMOylation of MORC2 at K767 attenuated the sensitivity of BC cells to DNA-damaging chemotherapeutic drugs, which may enhance the efficacy of chemotherapy in the treatment of MORC2-induced tumors by inhibiting its SUMOylation modification.^114^ Overall, high expression of MORC2 drives chemotherapy resistance to cancer. According to existing studies, some chemotherapeutic drugs and inhibitors can inhibit MORC2 expression and activity in multiple ways to improve the sensitivity of BC cells to chemotherapy.

## MORC2 in endocrine therapy

Abnormal activation of estrogen signaling is one of the main pathways of BC progression.^115^ Studies have shown that ER-positive BCs are more effectively treated with anti-estrogen therapy drugs such as tamoxifen (TAM) and fulvestrant (FUL) than ER-negative BCs, which can lead to endocrine therapy resistance.^116^, ^117^, ^118^ Meanwhile, TAM, FUL, and 17β-estradiol (E2) were found to enhance MORC2 stability in a GPER1-dependent manner. GPER1 can activate PRKAC that causes the phosphorylation of MORC2 at T582 and reduces its interaction with HSPA8 and LAMP2A to inhibit its CMA-mediated lysosomal degradation, which maintains MORC2 protein stabilization and resists endocrine therapy.^28^

## Conclusions and perspectives

Originally, MORC2 was identified as a chromatin modifier. At present, MORC2 has been considered an oncogene and an emerging cancer marker that promotes cancer cell proliferation,^27^^,^^30^^,^^81^ invasion,^76^^,^^77^^,^^104^ metastasis,^21^^,^^25^^,^^80^ and treatment resistance.^26^, ^27^, ^28^^,^^78^ Aberrant amplification and mutation of MORC2 are frequently shown in human multiple cancers.^24^^,^^25^^,^^33^ Thus, MORC2 has recently attracted a great deal of attention due to its important role in cancer. The current review analyzed several studies on MORC2 in human cancer and attempted to summarize its precise role in cancer development and treatment.

With the in-depth understanding and refinement of MORC2 literature research, we found that MORC2 holds a central position in regulating cancer progression and signaling pathways. For instance, some protein kinases and ncRNAs as upstream important regulators of MORC2 improve its activity or expression.^25^^,^^46^^,^^47^ Subsequently, activated or overexpressed MORC2 induces its oncogenic events and cancer hallmarks by regulating diverse downstream target genes,^18^^,^^75^^,^^78^ which covers its action in the growth, invasion, and metabolic programming of cancer cells, and its response to cancer therapy. Overexpression of MORC2 in cancer has been found to drive therapeutic resistance; however, future studies are needed to confirm whether targeted MORC2 inhibitors in combination with chemotherapeutic drugs, radiotherapy, or endocrinal therapy have synergistic effects on the overexpression of MORC2 in BC and other various cancers. In light of these, MORC2 may serve as a potential tumor biomarker and drug target, offering a novel candidate protein for tumor diagnosis and treatment.

Overall, MORC2 is considered a diagnostic biomarker and therapeutic target for various cancers, as well as a key factor in treatment resistance in some cancers. However, it is a long and tortuous process to achieve targeted MORC2 drugs and clinical transformation. Although some chemotherapeutic drugs and inhibitors can inhibit the expression and activity of MORC2 in various ways, including by disrupting its stability and inhibiting its ATPase activity and via the SUMOylation pathway to promote the sensitivity of BC cells to chemotherapy, the regulatory mechanism underlying MORC2 expression is not well understood. Nonetheless, we found that these findings were based on BC, and chemotherapeutic drugs used in these studies were also used to treat BC in the clinic. Therefore, chemotherapeutic drugs or inhibitors for the treatment of BC may not be suitable for other MORC2-driven tumors. Therefore, we believe that these strategies for blocking the expression and activity of MORC2 in BC cells warrant further investigation and verification in other cancer types. Thus, identifying and selecting the efficacy of these inhibitors is crucial to carry out the goal for clinical application. Importantly, suitable cancer patients who are screened out based on MORC2 overexpression may benefit from treatment with targeted MORC2 inhibitors for individualized therapy, which could resolve most of the existing challenges.

## Author contributions

GLW conceived, supervised and polished the manuscript. SZ and AYG performed writing and prepared figures. HW, JL, CSD and JYR drew figures and summed up the literature in the manuscript. All authors read and approved the final manuscript.

## Conflict of interests

The authors declare that they have no competing interests.

## Funding

This work was financially supported in part by grants from the 10.13039/501100001809National Natural Science Foundation of China (No. 81572611 and 81828009), the Foundation Committee of Basic Research of Liaoning Province, China (No. LJKMZ20221205), and the Application Foundation Plan Project of Liaoning Provincial Department of Science and Technology (China) (No. 2023JH2/101300012).

## Data availability

The dataset generated and/or analyzed during the current study is available in the cBioPortal (http://www.cbioportal.org/), Gene Expression Profiling Interactive Analysis (GEPIA) (http://gepia.cancer-pku.cn/), and PhosphoSitePlus repository (http://phosphosite.org/).
